# Our Food and Ourselves

**Published:** 1917-12-15

**Authors:** 


### The Hospital

The Workers' Newspaper of Administrative Medicine and Institutional Life, Administration, National Insurance and Health.

No. 1645, Vol. LXIII. SATURDAY, DECEMBER 15, 1917. PRICE ONE PENNY

### OUR FOOD AND OURSELVES.

British people since the war have grown so accustomed to have first one thing and then another placed under Government control, to the multiplying of Committees, Commissions, and all kinds of agencies charged to do much, which formerly was the individual's sole business and right, or to make us do necessary things for ourselves, until newspaper influence has tended to become less rather than greater, as the war progresses. Take the case of our food. Most strenuous efforts have been and are being made to awaken the inhabitants of the British Islands to patent facts, which the possessors of common sense amongst us must realise to be true and pressing. We have, for instance, two great facts which even children of intelligence must be beginning to realise. One is that the number of great ships being sunk by the U-boats cannot fail to limit many articles of food and other necessaries, which an island people, in the past, have been accustomed to have brought to their shores in unlimited quantities. Supplies of food varying in kind are, consequently, a limited quantity to-day, and now and then they have become so scarce as to render their purchase prohibitive by persons of small or ordinary income. It follows that every one of us should make it our pride and duty to cut down the consumption of food, and arrange its variety and nature in our own establishment—that is, in every house throughout the land—to proportions, which will help the Controller to keep the market equipped to the fullest possible extent, to provide food in sufficient quantities to maintain the health and vigour of all classes of the community.

Lord Rhondda, at a meeting he had with the Press on Monday, pressed these points upon his hearers, and asked every editor to do his best to make every one of the readers of a newspaper realise the truth, and do his duty to his family, himself, and the nation in the way we have indicated. If all follow this course none need starve, and everybody should enjoy vigorous health. To do less is to cease to be a patriot and to exhibit a measure of folly which will qualify the defaulter as a fit inmate for a lunatic asylum. The only reasons we can find, why all have not already put themselves and their families on rations, is, either a selfish indifference to their neighbours' claims, or the stupid feeling that, if rationing is necessary, the Government will impose it upon every individual amongst us. Do the latter appreciate that the Government might ration everybody to-morrow on the German system, but then a large majority must suffer, grievously, in many ways? Rationing in these islands, unfortunately, we must have, but Lord Rhondda, we are confident, is not the man to enforce it until he has got everything organised completely, and can guarantee that the incidence of the change will be as little onerous on the individual as possible. We hope we can claim that our readers have thoughtful brains and patriotic hearts, that with these facts before them they will adopt rations without delay, and make every effort to secure that all who come within their influence shall, promptly, do the same.

The second reason why rationing is essential and the pinch of inadequate supplies may yet reach everybody in these islands, if the war continues, is the admitted fact that the harvest this year has failed in some of the countries where our Allies reside, and that they have to be supplied with an unexpectedly large amount of foodstuffs, whereby an additional strain has been put upon the carrying trade of this and other nations. It is impossible for us to do less than secure, by all means in our power, an adequate supply of food for our Allies, as well as for ourselves. That all must agree.

Christmas is almost with us, and good-will to men is a living force with British people, which should be present this year to a fuller extent, if possible, than ever before. To-day the words have a more personal application, for every one of us is up against the duty of securing, to the fullest possible extent, that their families, their neighbours and themselves shall not want for food and sustenance, if human effort can prevent it—as Lord Rhondda declares it can, in God's mercy.

Those who are so self-centred and so ignorant, when possessed of sufficient means, to go on keeping a liberal table and spending money prodigally on food, without enforcing economy in their own household, and placing themselves and those under their control upon rations in loyal co-operation with the Food Controller, are, however unconsciously, acting as the enemy of the Allies and the imperiller of the British people as an Empire and Nation.

Again, mere considerations of health should prevent any self-respecting and sane member of the race from adopting the attitude some are credited with: "I am not going to deny myself anything that money can buy until I am assured and guaranteed that everybody else is already on rations, and doing his best for the nation and the Allies in regard to foodstuffs." It is tempting Providence for man or woman to hold such unworthy and dishonouring sentiments, and to practise them is to place themselves on a level with the brute creation, whose instincts throughout are greediness, and whose type is popularly denominated to be that of the pig. The end of the whole matter is this, as Lord Rhondda put it convincingly and plainly. The time has come when it is essential that all classes, and every individual member of the community, should awaken to the facts we have set forth in this article, and play the man and woman with a whole-hearted recognition of the situation, and a vitalising love of their country and their comrades.

Compulsory rationing will necessarily come the moment it can be organised on the lines we have indicated, but it is the privilege of free men and women—English and Scotch, Welsh and Irish—not to wait for this consummation. The plain duty of all is to realise the position, to make it their business to provide that he and she individually, and all under their control, are on such a basis, so far as eating and drinking are concerned, that when compulsory rationing arrives, it shall be their pride that not one of them will have to make any change whatever in home, family, or individual food arrangements. We have sufficient confidence in our readers and our race to believe that the truth, as we have received it from Lord Rhondda, will sink deep into the hearts of British people, to their own uplifting and the salvation of the nation.

